# Can Polymorphisms in NLRP3 Inflammasome Complex Be Associated with Postmenopausal Osteoporosis Severity?

**DOI:** 10.3390/genes13122271

**Published:** 2022-12-02

**Authors:** Werbson Lima Guaraná, Camilla Albertina Dantas Lima, Alexandre Domingues Barbosa, Sergio Crovella, Paula Sandrin-Garcia

**Affiliations:** 1 Keizo Asami Institute (iLIKA), Federal University of Pernambuco, Recife 50670-901, Pernambuco, Brazil; 2 Genetics Postgraduate Program, Federal University of Pernambuco, Recife 50670-901, Pernambuco, Brazil; 3 Division of Rheumatology, Clinical Hospital, Federal University of Pernambuco (UFPE), Recife 50740-900, Pernambuco, Brazil; 4 Biological Sciences Program, Department of Biological and Environmental Sciences, College of Arts and Sciences, Qatar University, Doha P.O. Box 2713, Qatar; 5 Department of Genetics, Federal University of Pernambuco, Recife 50670-901, Pernambuco, Brazil

**Keywords:** osteoporosis, NLRP3, inflammasome, single-nucleotide variants, bone mineral density, vitamin D

## Abstract

The immune system plays a critical role in bone homeostasis and, consequently, in the pathophysiology of postmenopausal osteoporosis (OP) since estrogen deficiency induces the inflammasome and increases production of pro-inflammatory cytokines, such as IL-1β and IL-18. NLRP3 inflammasome complex genes have been related with bone homeostasis in cellular and animal models. Here, we performed an association study evaluating SNVs (single-nucleotide variants) in inflammasome NLRP3 pathway genes (NLRP3, CARD8, CASP1, IL-18, and IL-1β) to assess whether variants in these genes could be related to susceptibility to primary OP in postmenopausal women. Methods: We genotyped 196 postmenopausal OP patients and 103 healthy controls using SNV-specific Taqman^®^ probes. Data and statistical analyses were performed using the SNPstats and GraphPad Prism 8 software. Results: We showed an association between NLRP3 rs35829419 CA genotype and lower bone mineral density (BMD) mean at the lumbar spine (*p* = 0.001); we also observed an association between IL-1β rs16944 AA genotype and higher BMD mean at the total hip (*p* = 0.009). The IL-1β rs16944 GG was associated with lower alkaline phosphatase levels (ALP) (*p* = 0.009), and the IL-18 rs1946519 AA was associated with lower vitamin D levels (*p* = 0.018). Additionally, OP patients presented deficient vitamin D and parathyroid hormone (PTH). Conclusions: The NLRP3 inflammasome complex SNVs were associated with OP severity, possibly indicating these genes’ participation in bone metabolism and its dysregulation.

## 1. Introduction

Osteoporosis (OP) is a degenerative osteometabolic disease characterized by an imbalance between the processes of bone formation, which leads to a progressive loss of bone mineral density (BMD) and deterioration of bone microarchitecture, increasing skeletal fragility and, consequently, the occurrence of fractures in OP patients [1]. With the progressive increase in population longevity, the prevalence of OP has risen significantly in recent years [2]. Consequently, it is estimated that around 30% of these patients die due to fractures or the development of morbidities and secondary diseases [3].

The immune system plays a critical role in bone metabolism and postmenopausal OP pathophysiology. For example, the estrogen deficiency observed in women in postmenopausal conditions induces both osteoclast differentiation and activity due to the higher expression of nuclear factor kappa-B ligand (RANKL) and other pro-osteoclastogenic cytokines, such as interleukin (IL)-1, IL-6, and tumor necrosis factor-α (TNF-α), in the bone marrow [4,5].

In this context, the inflammasome acts a cytoplasmic multi-protein complex capable of triggering an inflammatory cascade, induced by infectious agents or endogenous stimuli, that activates caspase-1 (CASP1), which is responsible for generating bioactive forms of IL-1β and IL-18 [6]. The complex assembly is initiated by nucleotide-binding domain and leucine-rich repeat receptors (NLRs) or absent in melanoma 2 (AIM2)-like receptors (ALRs) that mediate host recognition of pathogen-associated molecular patterns (PAMPs), which are released during bacterial, viral, fungal, and protozoan infections, or by damage-associated molecular patterns (DAMPs) released during cellular damage [7].

Additionally, nucleotide-binding oligomerization domain-like receptor family pyrin domain-containing 3 (NLRP3) is the best characterized inflammasome of the NLR family, consisting of the NLRP3 scaffold, the apoptosis-associated speck-like protein containing a CARD (ASC) adaptor, and CASP1 [7,8]. NLRP3 inflammasome is activated by various stimuli, including PAMPs and DAMPs. Currently, a two-signal mechanism for NLRP3 inflammasome activation in macrophages has been proposed [8,9]. The priming signal (Signal 1) occurs by priming stimuli of PAMPs or DAMPs, leading to activation of the transcription factor nuclear factor-κB (NF-κB) and subsequent upregulation of inflammasome-related genes NLRP3 and pro-IL-1β, which, unlike pro-IL-18, is not constitutively expressed in resting macrophages [9,10]. The activation signal (Signal 2) is triggered by a variety of stimuli following this priming step, including extracellular ATP, K+, pathogen-associated RNA, and bacterial and fungal toxins and components that induce the assembly of NLRP3, ASC, and pro-CASP1, leading to inflammasome activation [11,12]. Once activated, the adaptor molecule ASC, which also contains a caspase activation and recruitment domain (CARD) found in the effector enzyme pro-CASP1, is recruited, resulting in the cleavage of pro-CASP1 into CASP1. Activated CASP1 processes pro-IL-1β and pro-IL-18 into their mature forms and promotes their release in the extracellular environment. At the same time, the activated CASP1 fragment can induce cell pyroptosis [13].

Single-nucleotide variants (SNVs) in the inflammasome NLRP3 pathway (NLRP3 rs35829419; rs10754558; IL1β rs16944; IL18 rs1946519; CARD8 rs2043211; and CASP1 rs61751523) have been associated with an increase in inflammation and different diseases, including the autoinflammatory ones [14,15,16,17]. In this context, autoinflammatory diseases have been associated with bone destruction or arthritis due to the regulation that inflammatory responses exert in the process of bone remodeling [18]. For example, Snouwaert and colleagues [19] observed that mutation in the NLRP3 gene caused arthropathy and OP in an NLRP3 gene humanized mice model. On the other hand, Detzen and colleagues [4] observed that impaired skeletal development in knockout (Nlrp3−/−) mice resulted in a shorter stature than in Nlrp3+/+ mice. Furthermore, these growth defects were associated with altered femur bone growth, characterized by a deficient growth plate and an osteopenic profile of the trabeculae.

For its role in bone metabolism, we evaluated whether the SNVs known to have a functional impact on the NLRP3 inflammasome (NLRP3 rs35829419; rs10754558; CASP1 rs61751523; IL-1β rs16944; IL-18 rs1946519; and CARD8 rs2043211) were associated with postmenopausal women OP. Additionally, we assessed all patients’ serum bone remodeling markers, BMD, clinical features, and their relation to the SNVs investigated.

## 2. Materials and Methods

### 2.1. Subjects

This study included a total of 196 postmenopausal OP women and 103 postmenopausal control individuals. All patients were recruited from the Rheumatology Division at Clinical Hospital, Federal University of Pernambuco (UFPE), Recife, Pernambuco, Brazil. Menopause was defined according to the World Health Organization (WHO) criteria as amenorrhea for at least one year in women over 45 years old without any other pathology. The OP diagnosis followed the WHO criteria based on the T-score of the BMD measurement of the lumbar spine, femoral neck, and total hip, using dual-energy X-ray absorptiometry (DXA) [20].

Following the WHO criteria, the control group included postmenopausal women without a diagnosis of OP or osteopenia, according to DXA exam; without fracture history after menopause; and no secondary OP in medical history, physical examination, or laboratory tests. Additionally, no subjects from the study were on hormone replacement therapy. Individuals with cancer, diabetes, and other rheumatology diseases were excluded [21]. All follow-up information was obtained directly from medical records.

The clinical and biochemical bone markers’ levels were evaluated for both patients and control group: age at menarche (years), age at menopause (years), vitamin D (ng/mL), calcium (mg/dL), alkaline phosphatase (ALP; U/L), phosphorus (mg/dL), parathyroid hormone (PTH; pg/mL), and magnesium (mg/dL).

All the participants provided written informed consent approved by the local Research Ethics Committee (CEP/CCS/UFPE No. 513/11), following the rules of the 1964 Helsinki Declaration.

### 2.2. SNVs Selection and Genotyping

Genomic DNA was extracted from peripheral blood leukocytes using the Wizard genomic DNA purification kit (Promega, Madison, WI, USA), following the manufacturer’s guidelines. SNVs within NLRP3 (rs35829419; rs10754558), CASP1 (rs61751523), IL-1β (rs16944), IL-18 (rs1946519), and CARD8 (rs2043211) genes were selected according to previous studies [22,23], following the 10% minimum allele frequency (MAF) according to NCBI (National Center for Biotechnology Information). Genotyping was performed using specific fluorogenic allele-specific TaqMan probes (Applied Biosystems, Foster City, CA, USA) on a ABI7500 real-time PCR system (Thermo Fisher, Madison, WI, USA). In addition, 25 ng of DNA was amplified and genotyped using the following protocol: 95 °C for 10 s, 40 cycles 95 °C for 30 s, 60 °C for 90 s, and final cycle at 60 °C for 60 s for all TaqMan probes utilized. The probes’ specifications are available at https://products.appliedbiosystems.com/ (accessed on 24 October 2022) (Assays ID: NLRP3 C__25648615_10, C__26052028_10; CASP1 C__64657193_10; IL-1β C___1839943_10; Il-18 C___2898459_20; and CARD8 C__11708080_1_).

### 2.3. Statistical Analysis

Differences between the SNVs genotypic frequencies in OP patients and the control group were assessed using the chi-square test. Odds ratios (ORs) and 95% confidence intervals (CIs) were also calculated. Allelic and genotypic frequencies and Hardy–Weinberg equilibrium (HWE) were evaluated using the SNPStats tool (Available online: http://bioinfo.iconcologia.net/SNPstats (accessed on 24 October 2022). Differences between healthy control and OP groups were analyzed using student’s T or Mann–Whitney for parametric and non-parametric data, respectively. In the same way, ANOVA or Kruskal–Wallis tests, for parametric and non-parametric data, respectively, were performed to compare the genotypes frequencies related to BMD and biochemical markers levels. When a significant difference was observed, the Tukey or Dunn’s tests were applied for pairwise comparisons of genotypes. Associations between genotypes, demographic, and clinical quantitative variables were performed by multinomial logistic regression, in which genotypes were used as dependent variables and all the demographic and clinical as covariates. All statistical analyses were conducted using Graphpad Prism software version 8.0 (GraphPad Software, San Diego, CA, USA) and IBM SPSS statistic software version 26.0 (IBM Corp, Armonk, NY, USA). Differences were accepted significantly at *p*-values < 0.05.

## 3. Results

### 3.1. Clinical and Biochemical Bone Markers Levels

We assessed 196 patients with OP and 103 individuals in the control group. The demographical data comparison showed no statistically significant difference between age (OP: 62 years old; control: 61 years old, *p* = 0.06); however, a statistically significant difference was observed in the average of years since menopause, with OP patients presenting a mean of 16 years (range: 3–36), while the control group presented a mean of 13 years (range: 2–35) (*p* = 0.019). In the clinical data comparison, we observed statistically significant differences in biochemical markers. The OP patients presented lower levels of vitamin D than the control group (*p* = 0.001); OP patients also showed higher levels of serum PTH (*p* = 0.043). No significant difference was observed for: calcium, ALP, phosphorus, and magnesium (Table 1).

### 3.2. Genetic Association Study

#### 3.2.1. SNVs and OP Susceptibility

Inflammasome genes, SNVs, allele, and genotype frequencies were compared between OP and controls, with the aim of detecting a possible association with susceptibility to developing OP. Allelic and genotypic frequencies from the selected SNVs were in HWE in both OP patients and control individuals. Low or null frequencies were observed for the SNVs genotypes NLRP3 rs35829419 AA and rs10754558 GG, and CASP1 rs61751523 CC and CARD8 rs2043211 TT in both groups. Moreover, a borderline association with protection was observed for the IL-1β rs16944 genotype AG in the codominant model for OP susceptibility (OR = 0.53; CI = 0.29–0.98; *p* = 0.05). However, none of the hypothesized associations reached statistical significance. All genotype and allelic frequencies are shown in Table 2.

#### 3.2.2. SNVs and Correlation with BMD

**Figure 1:**
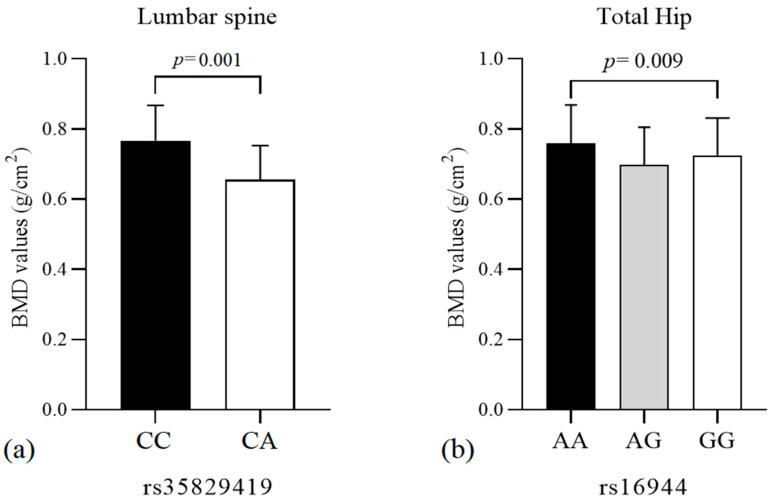
Comparison of bone mineral density stratified according to the genotypes of single-nucleotide variants rs35829419 of NLRP3 and rs16944 of IL-1β gene. (**a**) Comparison of bone mineral density of lumbar spine area between genotypes of rs35829419 of NLRP3. (**b**) Comparison of bone mineral density in the total hip between genotypes of rs16944 of the IL-1β gene. Statistical tests: (**a**) *p*-value student’s T = 0.001. (**b**) *p*-value ANOVA = 0.012; *p* Tukey post hoc test = 0.009 (AA vs. GG genotype).

Regarding BMD, the average levels (g/cm^2^) in regions measured by DXA exam (lumbar spine, femoral neck, and total hip) were compared to each polymorphism tested. We observed a statistically significant lower BMD mean of lumbar spine among patients for the NLRP3 SNV rs35829419 CA compared with CC (CC= 0.766 ± 0.10; CA = 0.655 ± 0.09 g/cm^2^, *p* = 0.001). Furthermore, we observed a statistically significant higher BMD mean of total hip in OP patients for the IL-1β SNV rs16944 AA when compared with GG (AA = 0.759 ± 0.10; AG = 0.697 ± 0.10; GG = 0.725 ± 0.10, *p* = 0.009) (Figure 1).

#### 3.2.3. SNVs and Biochemical Markers

**Figure 2:**
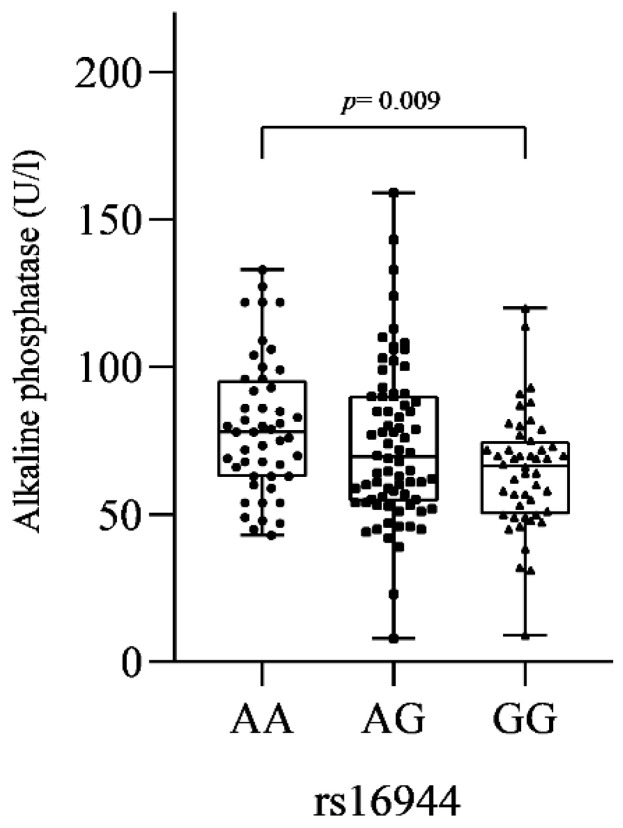
Comparison of alkaline phosphatase levels according to genotypes of single-nucleotide variant rs16944 in IL-1β gene. Statistical tests: *p*-value Kruskall–Wallis = 0.012; *p*-value Dunn’s post hoc test = 0.009 (AA vs. GG genotype).

In the comparison of serum biochemical markers between SNVs genotypes in our study populations (patients OP and control), the polymorphism IL-1β rs16944 showed a lower level of ALP serum in the GG genotype (AA = 79.70 ± 22.86; AG = 73.90 ± 26.69; GG = 64.78 ± 19.88 U/L), with a statistically significant difference when compared with AA (*p* = 0.009) (Figure 2).

**Figure 3:**
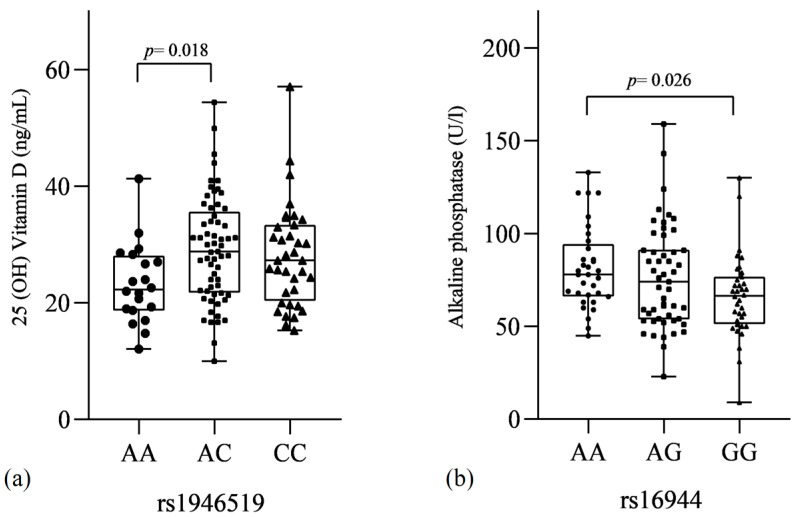
Comparison of biochemical markers of bone homeostasis in serum according to genotypes of single-nucleotide variants rs1946519 of IL-18 and rs1644 of IL-1β gene. (**a**) Comparison of 25(OH) Vitamin D levels between genotypes of rs1946519 of IL-18. (**b**) Comparison of Alkaline phosphatase levels between genotypes of rs16944 of IL-1β. Statistical tests values: (**a**) *p*-value ANOVA = 0.029; *p*-value Tukey = 0.018 (AA vs. AC); (**b**) *p*-value ANOVA/Kruskall–Wallis = 0.023; *p*-value Dunn’s test = 0.026 (AA vs. GG).

When compared within OP patients, lower levels of ALP were observed in IL-1β rs16944 GG (AA = 81.53 ± 22.04; AG = 76.28 ± 27.54; GG = 65.99 ± 22.14 U/L) (AA vs. GG *p* = 0.026). In addition, lower levels of vitamin D were also observed in IL18 rs1946519 AA subjects (AA = 23.26 ± 6.75; AC = 29.18 ± 9.08; CC = 28.05 ± 8.77 ng/mL) (AA vs. AC *p* = 0.018) (Figure 3). The other SNVs and serum biochemical markers did not show statistically significant differences. In the same way, multivariate analysis did not show statistically significant differences among genotypes and demographic and clinical features.

To summarize our results, despite the lack of association between the inflammasome genes’ SNVs and OP occurrence, a statistically significant correlation between genotypes and BMD levels was observed for SNVs rs35829419 CA of NLRP3 and rs16944 GG of IL-1β gene. Furthermore, lower PTH serum level was associated with the variant rs16944 GG in IL-1β gene, and lower vitamin D levels associated with IL-18 rs1946519 AA patients.

## 4. Discussion

In our study, we assessed the SNVs with the NLRP3 inflammasome pathway, rs35829419, rs10754558, CASP1 rs61751523, IL-18 rs1946519, IL-1β rs16944, and CARD8 rs2043211, and their role in OP susceptibility as well as in serum bone remodeling markers, BMD, and clinical features. We observed an association of NLRP3 rs35829419 and IL-1β rs16944 genotypes with BMD levels.

The NLRP3 rs35829419 variant is a C>A SNV located at exon 3 [24]. This variant is a gain-of-function mutation that leads to an increased production of IL-1β, which is associated with various inflammatory diseases such as rheumatoid arthritis, Crohn’s disease, and celiac disease [16,17]. The IL-1β binds macrophages’ receptors and promotes the generation of RANKL, which binds to RANK on osteoclast precursor cells, leading to osteoclasts activation. Therefore, IL-1β acts as an osteogenic inhibitor and bone resorption stimulator [25]. Additionally, in a previous study by Youm and colleagues [26], NLRP3 was reported as overexpressed in an aging mouse model. In contrast, the 24-month-old knockout (NLRP3−/−) mice had significantly higher bone mineral density and total bone area with significantly increased cortical and trabecular bone thickness. In our study, we observed that OP patients with rs35829419 CA genotype had lower BMD at the lumbar spine compared to CC patients (*p* = 0.001), according to the high bone resorption expected due to the presence of allele A.

As aforementioned, IL-1β increases with estrogen deficiency and plays an essential role in bone loss, thus stimulating the expression of the receptor activator of RANKL in osteoblasts, which can lead to a massive upregulation of osteoclasts and inhibition of osteoblasts. For example, the activation of osteoclasts via pro-inflammatory cytokines, such as IL-1β, in chronic inflammatory conditions leads to excessive systemic bone loss by upregulation of RANKL [25,27]. In this context, the IL-1 β rs16944 variant A>G is in the promoter region of the gene, which is possibly involved in the regulation of IL-1β [28,29]. Moreover, the SNV rs16944 G was associated with increased transcriptional activity and clinical conditions such as cardiovascular disease and gastric cancer [26]. Additionally, He and colleagues [30] investigated IL-1β genetic variants and predisposition to osteoporosis among the northwestern Chinese Han population. The authors observed an association of the IL-1 β rs16944 G allele with an elevated susceptibility to OP, especially in Chinese women >60 years (OR = 1.19, *p* = 0.037). These results are consistent with our findings, in which OP patients carrying the IL-1 β rs16944 AA genotype showed higher BMD at total hip compared to AG and GG (AA vs. GG *p* = 0.009), indicating a higher osteoclastic activity related to the G allele.

Regarding the biochemical markers and SNVs analysis results, lower serum ALP was observed in the presence of IL-1β rs16944 GG genotype in OP patients and controls. ALP is a marker of bone remodeling by osteoblast activity, so low serum ALP indicates reduced osteoblasts action or bone formation [31,32]. Therefore, the lower ALP level reinforces the observed association between SNV IL-1β rs16944 G-allele and low BMD. Additionally, serum vitamin D was lower in OP patients with IL-18 rs1946519 AA genotype (*p* = 0.018). The SNV IL-18 rs1946519 located at promoter position-607 of IL-18 gene (11q22.2–q23.3) has been reported as associated with the decrease of IL-18 [33]. Low vitamin D status is a risk factor for BMD loss in postmenopausal women, with deficiency relatively high among postmenopausal women and a provisional diagnosis of osteoporosis [34]. However, high inflammation is the common factor between diseases and low vitamin D concentrations [35]. Therefore, the presence of AA genotype could contribute to maintaining lower vitamin D levels.

When comparing OP patients and control groups, the OP patients present a lower serum vitamin D (28.15 ± 8.68, *p* = 0.001) and higher PTH (56.39 ± 27.25, *p* = 0.043). Estrogen has an essential role in increasing the activity of the enzyme responsible for activating vitamin D; therefore, declining estrogen levels during menopause could lead to vitamin D deficiency [36]. Additionally, both PTH and vitamin D are the two major regulators of mineral metabolism. They play critical roles in maintaining calcium and phosphate homeostasis and developing and maintaining bone health. They form a tightly controlled feedback cycle, PTH being a major stimulator of vitamin D synthesis in the kidney, while vitamin D exerts negative feedback on PTH secretion [37].

Finally, OP patients showed more time with menopause (16 years) than control group (13 years) (*p* = 0.019). This data agrees with the literature, where a longer time of menopause is associated with a higher risk of OP development due to more intense loss of bone mass over the years [38].

## 5. Conclusions

Our results suggest an influence of inflammasome SNVs on bone formation, biochemical markers levels, and OP severity. Furthermore, these findings highlight the importance of the inflammasome on bone homeostasis and OP prognostic. Being aware of the limitation of our study, related to the absence of functional validation, our simple genetic approach might contribute to better elucidating the mechanisms at the basis of postmenopausal OP development.

## Figures and Tables

**Table 1:** Demographic and clinical characteristics of patients with osteoporosis (OP) versus control group.

Characteristics	OP	Control	*p*
**Demographic**			
Age (range)	62 (51–72)	61 (53–73)	0.06
Mean of years since menopause (range)	16 (3–36)	13 (2–35)	0.019 *
**Clinical**			
Vitamin D (ng/mL)	28.15 ± 8.68	32.75 ± 10.72	0.001 *
Calcium (mg/dL)	9.45 ± 0.67	9.48 ± 0.78	0.79
Alkaline phosphatase (U/L)	75.48 ± 28.38	70.16 ± 23.70	0.33
Phosphorus (mg/dL)	3.47 ± 0.56	3.61 ± 0.58	0.11
Parathyroid hormone (pg/mL)	56.39 ± 27.25	48.33 ± 27.05	0.043 *
Magnesium (mg/dL)	2.06 ± 0.26	2.02 ± 0.47	0.74

Demographic quantitative variables were expressed as mean and min-max. Clinical quantitative were expressed as mean and standard deviation. * Statistically significant.

**Table 2:** Allele and genotype frequencies between OP patients and controls in SNVs of NLRP3 inflammasome.

SNVs	Model	Alleles/Genotypes	Controls, *n* (%)	OP, *n* (%)	*p*	OR	95% CI
NLRP3 rs35829419		C	196 (97)	364 (97)		1	
		A	6 (3)	10 (3)	0.83	0.90	0.30–2.46
	Codominant	CC	95 (94)	177 (95)		1	
		CA	6 (6)	10 (5)	0.83	0.89	0.33–2.52
		AA	0 (0)	0 (0)	ND	ND	ND
	HWE		1	1			
NLRP3 rs10754558		G	70 (36)	136 (36)		1	
		C	126 (64)	246 (64)	0.97	1.00	0.70–1.44
	Codominant	GG	9 (9)	25 (13)		1	
		GC	52 (53)	86 (45)	0.22	0.59	0.24–1.35
		CC	37 (38)	80 (42)	0.56	0.77	0.54–3.09
	Dominant	GG	9 (9)	25 (13)		1	
		GC-CC	89 (81)	166 (87)	0.75	0.67	0.29–1.52
	Recessive	GG-GC	61 (62)	111 (58)		1	
		CC	37 (38)	80 (42)	1.12	1.18	0.71–1.96
	Log-additive	---	---	---	0.98	0.99	0.69–1.44
	HWE		0.19	0.87			
CASP1 rs61751523		T	140 (92)	283 (94)		1	
		C	12 (8)	17 (6)	0.36	0.70	0.33–1.50
	Codominant	TT	64 (84)	134 (89)		1	
		TC	12 (16)	15 (10)	0.21	0.59	0.27–1.39
		CC	0 (0)	1 (1)	0.49	ND	ND
	Dominant	TT	64 (84)	134 (89)		1	
		TC-CC	12 (16)	16 (11)	0.28	0.63	0.29–1.46
	Recessive	TT-TC	76 (100)	149 (99)		1	
		CC	0(0)	1 (1)	>0.99	ND	ND
	Log-additive	---	---	---	0.37	0.70	0.33–1.51
	HWE		1	0.38			
IL-18 rs1946519		A	79 (40)	144 (46)		1	
		C	121 (60)	172 (54)	0.17	0.77	0.54–1.12
	Codominant	AA	15 (15)	33 (21)		1	
		AC	49 (49)	78 (49)	0.36	0.72	0.35–1.42
		CC	36 (36)	47 (30)	0.17	0.59	0.28–1.28
	Dominant	AA	15 (15)	33 (21)		1	
		AC-CC	85 (85)	125 (79)	0.25	0.66	0.34–1.28
	Recessive	AA-AC	64 (64)	111 (70)		1	
		CC	36 (36)	47 (30)	0.33	0.75	0.45–1.27
	Log-additive	---	---	---	0.17	1.28	0.89–1.84
	HWE		1	1			
IL-1β rs16944		A	96 (47)	189 (50)		1	
		G	110 (53)	189 (50)	0.43	0.87	0.62–1.23
	Codominant	AA	20 (20)	54 (29)		1	
		AG	56 (54)	81 (43)	0.05	0.53	0.29–0.98
		GG	27 (26)	54 (28)	0.39	0.74	0.36–1.45
	Dominant	AA	20 (20)	54 (29)		1	
		AG-GG	83 (80)	135 (71)	0.09	0.60	0.33–1.07
		Recessive	AA-AG	76 (74)	135 (72)		1	
	GG	27 (26)	54 (28)	0.68	1.12	0.64–1.89
	Log-additive	---	---	---	0.45	1.14	0.82–1.58
	HWE		0.43	0.06			
CARD8 rs2043211		A	145 (72)	275 (72)		1	
		T	55 (28)	105 (28)	0.97	1.00	0.69–1.46
	Codominant	AA	51 (51)	99 (52)		1	
		AT	43 (43)	77 (41)	0.75	0.92	0.56–1.52
		TT	6 (6)	14 (7)	0.72	1.20	0.45–3.28
	Dominant	AA	51 (51)	99 (52)		1	
		AT-TT	49 (49)	91 (48)	0.90	0.95	
	Recessive	AA-AT	94 (94)	176 (93)		1	
		TT	6 (6)	14 (7)	0.80	1.24	0.49–3.26
	Log-additive	---	---	---	0.97	1.01	0.68–1.49
	HWE		0.62	1			

OP, osteoporosis; HWE, Hardy–Weinberg equilibrium; *p*, chi-square test *p*-value; OR, odds ratio; CI, confidence interval; --- not applicable; ND, not determined.

## Data Availability

Not applicable.

## References

[1] Cummings S.R., Melton L.J. (2002). Osteoporosis I: Epidemiology and outcomes of osteoporotic fractures. Lancet.

[2] Shen Y., Huang X., Wu J., Lin X., Zhou X., Zhu Z., Pan X., Xu J., Qiao J., Zhang T. (2022). The Global Burden of Osteoporosis, Low Bone Mass, and Its Related Fracture in 204 Countries and Territories, 1990–2019. Front. Endocrinol..

[3] Lewiecki E.M., Wright N.C., Curtis J.R., Siris E., Gagel R.F., Saag K.G., Singer A.J., Steven P.M., Adler R.A. (2017). Hip fracture trends in the United States, 2002 to 2015. Osteoporos. Int..

[4] Detzen L., Cheat B., Besbes A., Hassan B., Marchi V., Baroukh B., Lesieur J., Sadoine J., Torrens C., Rochefort G. (2020). NLRP3 is involved in long bone edification and the maturation of osteogenic cells. J. Cell. Physiol..

[5] Gross O., Thomas C.J., Guarda G., Tschopp J. (2011). The inflammasome: An integrated view. Immunol. Rev..

[6] Evavold C.L., Kagan J.C. (2019). Inflammasomes: Threat-Assessment Organelles of the Innate Immune System. Immunity.

[7] Schroder K., Tschopp J. (2010). The Inflammasomes. Cell.

[8] Man S.M., Kanneganti T.D. (2015). Regulation of inflammasome activation. Immunol. Rev..

[9] Kelley N., Jeltema D., Duan Y., He Y. (2019). The NLRP3 Inflammasome: An Overview of Mechanisms of Activation and Regulation. Int. J. Mol. Sci..

[10] Bauernfeind F.G., Horvath G., Stutz A., Alnemri E.S., Macdonald K.L., Speert D.P., Fernandes-Alnemri T., Wu J., Monks B.G., Fitzgerald K. (2009). Cutting Edge: NF-κB Activating Pattern Recognition and Cytokine Receptors License NLRP3 Inflammasome Activation by Regulating NLRP3 Expression. J. Immunol..

[11] Murakami T., Nakaminami Y., Takahata Y., Hata K., Nishimura R. (2022). Activation and Function of NLRP3 Inflammasome in Bone and Joint-Related Diseases. Int. J. Mol. Sci..

[12] Netea M.G., Nold-Petry C.A., Nold M.F., Joosten L.A.B., Opitz B., van der Meer J.H.M., van de Veerdonk F.L., Ferwerda G., Heinhuis B., Devesa I. (2009). Differential requirement for the activation of the inflammasome for processing and release of IL-1β in monocytes and macrophages. Blood.

[13] Fusco R., Siracusa R., Genovese T., Cuzzocrea S., Di Paola R. (2020). Focus on the Role of NLRP3 Inflammasome in Diseases. Int. J. Mol. Sci..

[14] Valencia B.M., Cvejic E., Vollmer-Conna U., Hickie I.B., Wakefield D., Li H., Pedergnana V., Rodrigo C., Lloyd A.R. (2021). The severity of the pathogen-induced acute sickness response is affected by polymorphisms in genes of the NLRP3 inflammasome pathway. Brain Behav. Immun..

[15] Verma D., Särndahl E., Andersson H., Eriksson P., Fredrikson M., Jönsson J.-I., Lerm M., Söderkvist P. (2012). The Q705K Polymorphism in NLRP3 Is a Gain-of-Function Alteration Leading to Excessive Interleukin-1β and IL-18 Production. PLoS ONE.

[16] Wu Z., Wu S., Liang T. (2021). Association of NLRP3 rs35829419 and rs10754558 Polymorphisms with Risks of Autoimmune Diseases: A Systematic Review and Meta-Analysis. Front. Genet..

[17] Zhang Q., Fan H., Zhang J., Wang Y., Xing H. (2015). NLRP3 rs35829419 polymorphism is associated with increased susceptibility to multiple diseases in humans. Genet. Mol. Res..

[18] Yu C., Zhang C., Kuang Z., Zheng Q. (2021). The Role of NLRP3 Inflammasome Activities in Bone Diseases and Vascular Calcification. Inflammation.

[19] Snouwaert J.N., Nguyen M., Repenning P.W., Dye R., Livingston E.W., Kovarova M., Moy S.S., Brigman B.E., Bateman T.A., Ting J.P.-Y. (2016). An NLRP3 Mutation Causes Arthropathy and Osteoporosis in Humanized Mice. Cell Rep..

[20] Camacho P.M., Petak S.M., Binkley N., Diab D.L., Eldeiry L.S., Farooki A., Harris S.T., Hurley D.L., Kelly J., Lewiecki E.M. (2020). American Association of Clinical Endocrinologists/American College of Endocrinology Clinical Practice Guidelines for the Diagnosis and Treatment of Postmenopausal Osteoporosis—2020 Update. Endocr. Pract..

[21] Kanis J.A. (2008). Assessment of Osteoporosis at the Primary Health Care Level.

[22] Pontillo A., Brandao L., Guimaraes R., Segat L., Araujo J., Crovella S. (2010). Two SNPs in *NLRP3* gene are involved in the predisposition to type-1 diabetes and celiac disease in a pediatric population from northeast Brazil. Autoimmunity.

[23] Addobbati C., da Cruz H.L.A., Adelino J.E., Ramos A.L.M.T., Fragoso T.S., Domingues A., Duarte L.B.P., Oliveira R.D.R., Louzada-Júnior P., Donadi E.A. (2017). Polymorphisms and expression of inflammasome genes are associated with the development and severity of rheumatoid arthritis in Brazilian patients. Inflamm. Res..

[24] Theodoropoulou K., Spel L., Zaffalon L., Delacrétaz M., Martinon F. (2022). NLRP3 leucine-rich repeats control induced and spontaneous inflammasome activation in cryopyrin-associated periodic syndrome. J. Allergy Clin. Immunol..

[25] Jiang N., An J., Yang K., Liu J., Guan C., Ma C., Tang X. (2021). NLRP3 Inflammasome: A New Target for Prevention and Control of Osteoporosis?. Front. Endocrinol..

[26] Youm Y.-H., Grant R.W., McCabe L.R., Albarado D.C., Nguyen K.Y., Ravussin A., Pistell P., Newman S., Carter R., Laque A. (2013). Canonical Nlrp3 Inflammasome Links Systemic Low-Grade Inflammation to Functional Decline in Aging. Cell Metab..

[27] Pietschmann P., Mechtcheriakova D., Meshcheryakova A., Föger-Samwald U., Ellinger I. (2015). Immunology of Osteoporosis: A Mini-Review. Gerontology.

[28] Iacoviello L., Di Castelnuovo A., Gattone M., Pezzini A., Assanelli D., Lorenzet R., Del Zotto E., Colombo M., Napoleone E., Amore C. (2005). Polymorphisms of the interleukin-1β gene affect the risk of myocardial infarction and ischemic stroke at young age and the response of mononuclear cells to stimulation in vitro. Arterioscler. Thromb. Vasc. Biol..

[29] Rogus J., Beck J.D., Offenbacher S., Huttner K., Iacoviello L., Latella M.C., De Gaetano M., Wang H.-Y., Kornman K.S., Duff G.W. (2008). IL1B gene promoter haplotype pairs predict clinical levels of interleukin-1β and C-reactive protein. Qual. Life Res..

[30] He Z., Sun Y., Wu J., Xiong Z., Zhang S., Liu J., Liu Y., Li H., Jin T., Yang Y. (2020). Evaluation of genetic variants in *IL-1B* and its interaction with the predisposition of osteoporosis in the northwestern Chinese Han population. J. Gene Med..

[31] Roudsari J.M., Mahjoub S. (2012). Quantification and comparison of bone-specific alkaline phosphatase with two methods in normal and paget’s specimens. Casp. J. Intern. Med..

[32] Silva B.C., Bilezikian J.P. (2015). Parathyroid hormone: Anabolic and catabolic actions on the skeleton. Curr. Opin. Pharmacol..

[33] Giedraitis V., He B., Huang W.-X., Hillert J. (2001). Cloning and mutation analysis of the human IL-18 promoter: A possible role of polymorphisms in expression regulation. J. Neuroimmunol..

[34] Suganthan N., Kumanan T., Kesavan V., Aravinthan M., Rajeshkannan N. (2020). Vitamin D status among postmenopausal osteoporotic women: A hospital based cross-sectional study from Northern Sri Lanka. BMC Nutr..

[35] Mangin M., Sinha R., Fincher K. (2014). Inflammation and vitamin D: The infection connection. Inflamm. Res..

[36] LeBlanc E.S., Desai M., Perrin N., Wactawski-Wende J., Manson J.A.E., Cauley J.A., Michael Y.L., Tang J., Womack C., Song Y. (2014). Vitamin D levels and menopause-related symptoms. Menopause.

[37] Khundmiri S.J., Murray R.D., Lederer E. (2016). PTH and Vitamin, D. Compr. Physiol..

[38] Finkelstein J.S., Brockwell S.E., Mehta V., Greendale G.A., Sowers M.R., Ettinger B., Lo J.C., Johnston J.M.A., Cauley J., Danielson M.E. (2008). Bone Mineral Density Changes during the Menopause Transition in a Multiethnic Cohort of Women. J. Clin. Endocrinol. Metab..

